# Low reduction of HCV-RNA level at week 4 in HIV-1 infected patients with acute HCV Infection

**DOI:** 10.1186/1742-4690-9-S1-P54

**Published:** 2012-05-25

**Authors:** A Vincent, S Jonckheere, B Kabamba, JC Yombi, D Wilmes, L Belkhir, B Vandercam

**Affiliations:** 1 Internal Medicine - Infectious Dieases at Cliniques Universitaires Saint Luc, Bruxelles, Bruxelles, Belgium

## Introduction

The epidemic of acute Hepatitis C-virus infection (AHC) is an on-going issue, especially among MSM patients. Spontaneous HCV clearance rates have been estimated to be between 5-40%, with lower rates among HIV-1 co-infected patients. High rates of sustained virological response have been observed (60-80%) when patients are treated within 24 weeks of AHC diagnosis. Moreover, a spontaneous 2log reduction in HCV-RNA might be a good predictor of spontaneous HCV-RNA clearance in HIV-1 co-infected patients. The aim of this study is to analyse the incidence of 2-log-HCV-RNA spontaneous reduction in AHC in HIV-1 infected patients.

## Materials and method

We reviewed medical files of 930 HIV-1-infected patients from our Reference Centre. All patients diagnosed with AHC in 2010-2011 were included. Diagnosis of AHC was based on seroconversion and ALT elevation. HCV-RNA PCR data was collected at time of and on week 4 after diagnosis.

## Results

Eleven patients were included, but only 10 met our diagnostic criteria. One patient was excluded because of missing data. Demographic and clinical data are summarised in Table 1. All patients were MSM. All but 2 patients had genotype 1a HC-virus. On week 4, 3 patients presented with a spontaneous 2log HCV-RNA reduction. All were diagnosed with genotype 1a AHC.

**Table 1:** Demographic data (data shown in numbers (%) or median)

Population	
Male sex	9 (100%)
Age (median)	44 y
BMI (median)	23
Transmission risk-factor; MSM	9 (100%)
Ethnicity (Caucasian/Black)	9 (100%)

**HIV results**	

CD4-cells count (median)	565
HIV-RNA (copies/mL) (median)	<37
HAART+	9 (100%)

**HCV**	

Genotype 1a	7 (78%)
Genotype 4	2 (22%)
HCV-RNA To (copies/mL)	1049740
HCV-RNA T+4weeks (copies/mL)	552042
- 2 log at T+ 4w	3 (33%)
- 2 log at T+ 4w according to genotype	3 (100%) genotype 1a / 0 genotype 4

**Clinical features**	

AST (IU/L) T0 (median)	357
ALT (IU/L) T0 (median)	654
ALT (IU/L) T+4w (median)	131

## Conclusion

Although completed on a small number of patients, this audit suggests that only a low proportion (33%) of HIV-1 infected patients presenting with AHC will meet a favourable 2log-HCV-RNA-drop criteria. This is slightly lower than rates reported by NEAT (i.e. 48%). It implies that these co-infected patients should benefit from a close follow-up in order to start treatment within 24 weeks of AHC diagnosis, whenever it is needed and available.

